# Multi‐Valent Cation Strategies for Controlling Interphase Chemistry at the Lithium Metal Anode

**DOI:** 10.1002/smtd.202501449

**Published:** 2025-09-18

**Authors:** Peng Yan, Rui Xu, Matthias Weiling, Bixian Ying, Marian Cristian Stan, Christian Wölke, Masoud Baghernejad, Jia‐Qi Huang, Martin Winter, Peter Bieker, Isidora Cekic‐Laskovic

**Affiliations:** ^1^ Helmholtz‐Institute Münster (IMD‐4) Forschungszentrum Jülich GmbH Corrensstraße 48 48149 Münster Germany; ^2^ MEET Battery Research Center University of Münster Corrensstraße 46 48149 Münster Germany; ^3^ Advanced Research Institute of Multidisciplinary Science Beijing Institute of Technology Beijing 100081 China

**Keywords:** electric double layer, Li metal anode, multi‐valent cation, solid electrolyte interphase

## Abstract

The effectiveness of a solid electrolyte interphase (SEI) in lithium metal batteries (LMBs) is crucial for the reversible deposition and dissolution of lithium (Li). Herein, a multi‐valent cation (MVC) is proposed approach to enable superior LMB performance without increasing conducting salt concentration, thus reducing the cost and environmental footprint of LMBs. In this approach, a minimal amount of magnesium carbonate (MgCO_3_) of 0.05 m is added to a lithium hexafluorophosphate (LiPF_6_) based electrolyte, which effectively scavenges hydrogen fluoride (HF) generated from hydrolysis of LiPF_6_. Concurrently, the HF scavenging process dissolves MgCO_3_ microparticles and releases Mg^2+^ cations. It is noteworthy that multivalent Mg^2+^ cations, due to their high charge density, enrich the electric double layer with anions that preferentially decompose to form an anion‐derived SEI. Consequently, the MVC approach facilitates enhanced reversibility of Li metal deposition and dissolution, as well as stable galvanostatic cycling of LiNi_0.8_Mn_0.1_Co_0.1_O_2_ (NMC811)||Li cells. This approach provides a highly effective pathway for designing anion‐derived SEI, offering new insights into the control of Li metal interfaces.

## Graphical abstract

A multivalent‐cation strategy using suspension MgCO_3_‐containing electrolyte regulates the electric double layer to promote anion‐derived SEI formation on Li metal. CO_3_^2‐^ scavenges HF while Mg^2+^ enriches anions in the interphase, enabling dense Li deposits, reduced overvoltage, and extended cycle life of NMC811||Li cells with minimal, low‐cost additive loading.

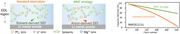

## 1 Introduction

Lithium metal is often referred to as the “holy grail” electrode material due to its low standard reduction potential (−3.04 V vs standard hydrogen electrode (SHE)) and high theoretical specific capacity (3860 mAh g^−1^).^[^ ^1^, ^2^ ^]^ Therefore, rechargeable lithium metal batteries (LMBs), which offer high energy density, have recently regained enormous attention in the growing markets for mobile devices and electric vehicles.^[^ ^3^ ^]^ However, the inhomogeneous Li metal deposition during repetitive charging and discharging results in the formation of high surface area lithium (HSAL), which consumes the electrolyte and leads to dendrite formation or even “dead lithium.”^[^ ^4^, ^5^ ^]^ This results in a short circuit and potentially leads to fire or explosion, posing a substantial barrier to the commercialization of LMBs.^[^ ^6^ ^]^ Extensive research has focused on modifying the solid electrolyte interphase (SEI), which strongly affects the Li metal deposition and dissolution processes and overall galvanostatic cycling performance.^[^ ^6^ ^]^ The SEI forms spontaneously upon contact between Li metal and the electrolyte, and its properties depend considerably on the nature of the decomposition products, which are closely related to the electrolyte composition.^[^ ^7^ ^]^ Organic carbonate‐based electrolytes, commonly used in commercial lithium‐ion batteries (LIBs), are by far not the best choice for liquid electrolyte LMBs.^[^ ^8^ ^]^

Recent research has indicated that an anion‐derived SEI outperforms a solvent‐derived SEI in inhibiting dendrite formation and growth, as well as related side reactions.^[^ ^9^ ^]^ To achieve an anion‐derived SEI, researchers have focused on regulating the solvation structure by either increasing the anion‐to‐solvent ratio or reducing the solvating capability of the solvent.^[^ ^10^, ^11^, ^12^ ^]^ Increasing the concentration of conducting salts, as in high‐concentration electrolytes (HCEs), decreases the proportion of free molecules. This allows anions to be more involved in the solvation sheath of Li^+^ ions, forming solvation clusters such as contact ion pairs (CIPs) or ion aggregates (AGGs). Such solvation structures enable anions to participate in the electric double layer (EDL), resulting in anion‐derived SEI. However, a large amount of lithium salt increases cost and reduces Li^+^ ion mobility, making practical application challenging. An alternative approach involves using non‐polar solvents with low solvating capability to promote CIPs and AGGs without increasing the concentration of conducting salt. However, options for weakly solvating solvents are limited, and a systematic screening process is needed to identify suitable solvents that balance the solvation and dissolution ability of Li conducting salts. Another way to promote anion‐derived SEI is to regulate the inner Helmholtz layer (IHL) with multi‐valent cations (MVCs). Divalent cations have a higher charge than Li^+^ions, strengthening anion coordination in the bulk electrolyte and recruiting anion‐cation complexes into the IHL, where they are preferentially reduced ahead of solvent species.^[^ ^13^, ^14^, ^15^ ^]^ Zhang et al. use Cu^2+^ ions to promote the dissolution of lithium nitrate (LiNO_3_) due to their stronger interaction with NO_3_^−^ than that of Li^+^.^[^ ^15^ ^]^ As a result, Cu^2+^‐NO_3_^−^ complexes are preferentially adsorbed and reduced to form the SEI.

Herein, we report an alternative approach for forming anion‐derived SEI using an MVC strategy. Incorporating a small amount of MgCO_3_ serves not only as a hydrofluoric acid (HF) scavenger but also allows the accumulation of anions in the EDL due to the presence of the multi‐valent Mg^2+^ cation. These accumulated anions within the EDL alter the interface properties, affecting the interphase formation and resulting in the formation of an anion‐derived SEI. This modified SEI facilitates homogeneous Li deposits and leads to a higher Coulombic efficiency (CE) in Li||Cu cells compared to the baseline electrolyte analogue. Moreover, the beneficial effects of MgCO_3_ have been demonstrated in a full cell setup with Li metal anode and Ni‐rich cathode, resulting in a prolonged lifespan.

## 2 Results and Discussion

### 2.1 Electrochemical Performance and Surface Characterization

**Figure 1:**
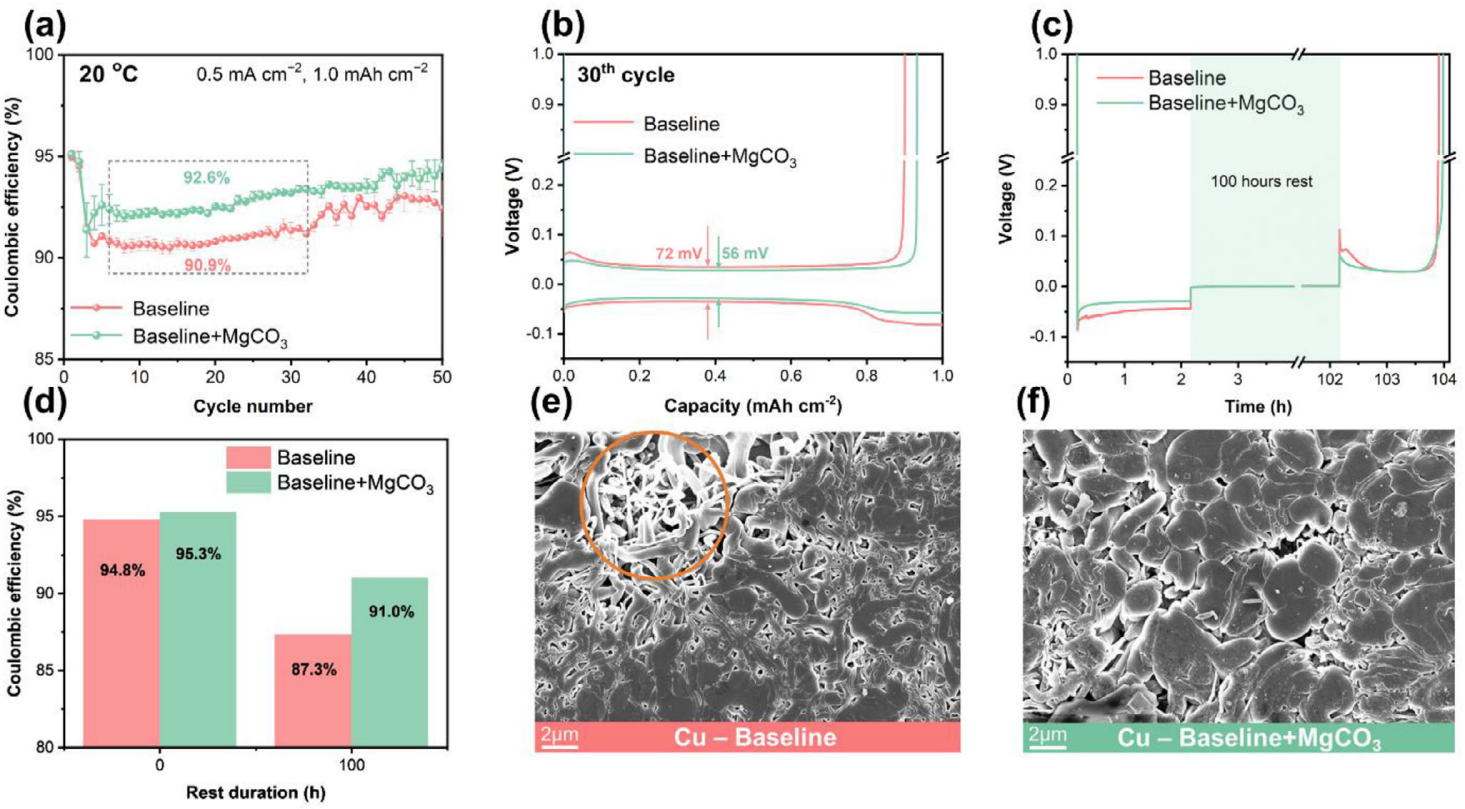
Electrochemical and morphological analysis of Li||Cu, cells with considered electrolytes. a) Coulombic efficiency of Li||Cu cells over the first 50 cycles. b) Voltage‐capacity profiles of the 30^th^ cycle in Li||Cu cells. c) Voltage‐time profile in Li||Cu cells with a 100 h rest applied between the Li metal deposition and dissolution processes, and d) corresponding CE. SEM images of the Cu surface harvested after 1^st^ Li deposition from Li||Cu cells using e) Baseline electrolyte and f) Baseline+MgCO_3_ electrolyte.

To verify the feasibility of using the MgCO_3_‐containing electrolyte in LMBs, two electrolyte formulations were considered: the baseline electrolyte (1M lithium hexafluorophosphate (LiPF_6_) in fluoroethylene carbonate (FEC): dimethyl carbonate (DMC) 1:4 by volume ratio) and the MgCO_3_‐containing electrolyte (baseline+0.05 m MgCO_3_). The Li||Cu cells were assembled to evaluate the CE of Li deposition and dissolution under conditions of 0.5 mA cm^−2^ current rate and 1.0 mAh cm^−2^ capacity. Initially, the CEs of both cells containing the two electrolytes were similar, with the cells containing MgCO_3_ enabling a slightly higher CE. However, a noticeable discrepancy was observed after 3 cycles, as the cell containing MgCO_3_ in the electrolyte formulation consistently provided a considerably higher CE compared to the baseline electrolyte counterpart (**Figure** **1**a). It was observed that cells with the baseline electrolyte achieved a CE of over 90% during galvanostatic cycling, which could be attributed to the effective SEI formation properties of FEC.^[^ ^16^ ^]^ However, fluctuations in CE were observed during further cycles for Li||Cu cells with baseline electrolytes. In contrast, cells with an electrolyte containing MgCO_3_ showed stable CE, indicating a beneficial impact of MgCO_3_ on the Li metal deposition and dissolution processes. This positive effect on the reversibility of Li metal deposition and dissolution is further evidenced by the voltage‐capacity profiles at selected cycle numbers (Figure 1b). Compared to baseline electrolytes, those with 0.05 m MgCO_3_ exhibited reduced polarization of 56 mV at the 30^th^ cycle, as opposed to the 72 mV observed with the baseline counterpart. Such reduced polarization may be related to a different type of SEI or the morphology of the accumulated Li on the Cu electrode.^[^ ^17^ ^]^ An additional experiment involved Li||Cu cells with a 100 h rest period between the Li deposition and dissolution processes to assess the stability of surface passivation against Li galvanic corrosion.^[^ ^18^ ^]^ Given that Li has the lowest standard redox potential, it reacts immediately upon contact with electrolytes and forms an SEI on the Li metal surface, which can be considered as chemical corrosion.^[^ ^1^ ^]^ However, galvanic corrosion occurs simultaneously when Cu serves as the substrate for Li metal deposition and both materials are in contact with the electrolyte.^[^ ^19^ ^]^ Galvanic corrosion can be accelerated by an unbalanced areal exposure to the electrolyte, such as with inhomogeneous or needle‐like deposited Li.^[^ ^20^, ^21^ ^]^ Figure 1c shows the voltage‐time profile of Li||Cu cells with baseline and MgCO_3_‐containing electrolytes. After a 100 h rest period, a substantial overvoltage was observed in cells with the baseline electrolyte compared to those with MgCO_3_. Additionally, the CE after the 100 h rest period was determined by dividing the capacity from Li dissolution after the rest period by the capacity from the initial Li deposition (Figure 1d). The addition of 0.05 m MgCO_3_ resulted in higher CE retention after 100 h rest (91.0% for baseline+MgCO_3_ vs 87.3% for baseline), indicating reduced galvanic corrosion in deposited Li, which most likely arose from a more passivating SEI chemistry as well as a more homogeneous and dense Li morphology.^[^ ^22^ ^]^

The morphology of Li metal deposited on Cu substrates harvested from Li||Cu cells after 1^st^ Li deposition was observed by scanning electron microscopy (SEM) (Figure 1e,f). Cells with baseline electrolyte displayed inhomogeneous, needle‐like Li deposits with a high surface area, which is a common characteristic of Li deposits formed in organic carbonate‐based solvent(s). In contrast, homogeneous, dense, and nodular Li deposits were observed in the MgCO_3_‐containing counterparts. The denser Li deposits with a lower surface area obtained with the baseline+MgCO_3_, as opposed to the baseline electrolyte, explain the improvement in CE and the reduction in overpotential of the resulting cells. Furthermore, the distinct morphologies of the Li deposits align with differing behaviors in galvanic corrosion, indicating a correlation between morphology and electrochemical stability. Ultimately, incorporating 0.05 m MgCO_3_ into the electrolyte resulted in homogeneous and nodular Li deposits that effectively suppressed the galvanic corrosion and overvoltage, enhancing CE in Li||Cu cells.

**Figure 2:**
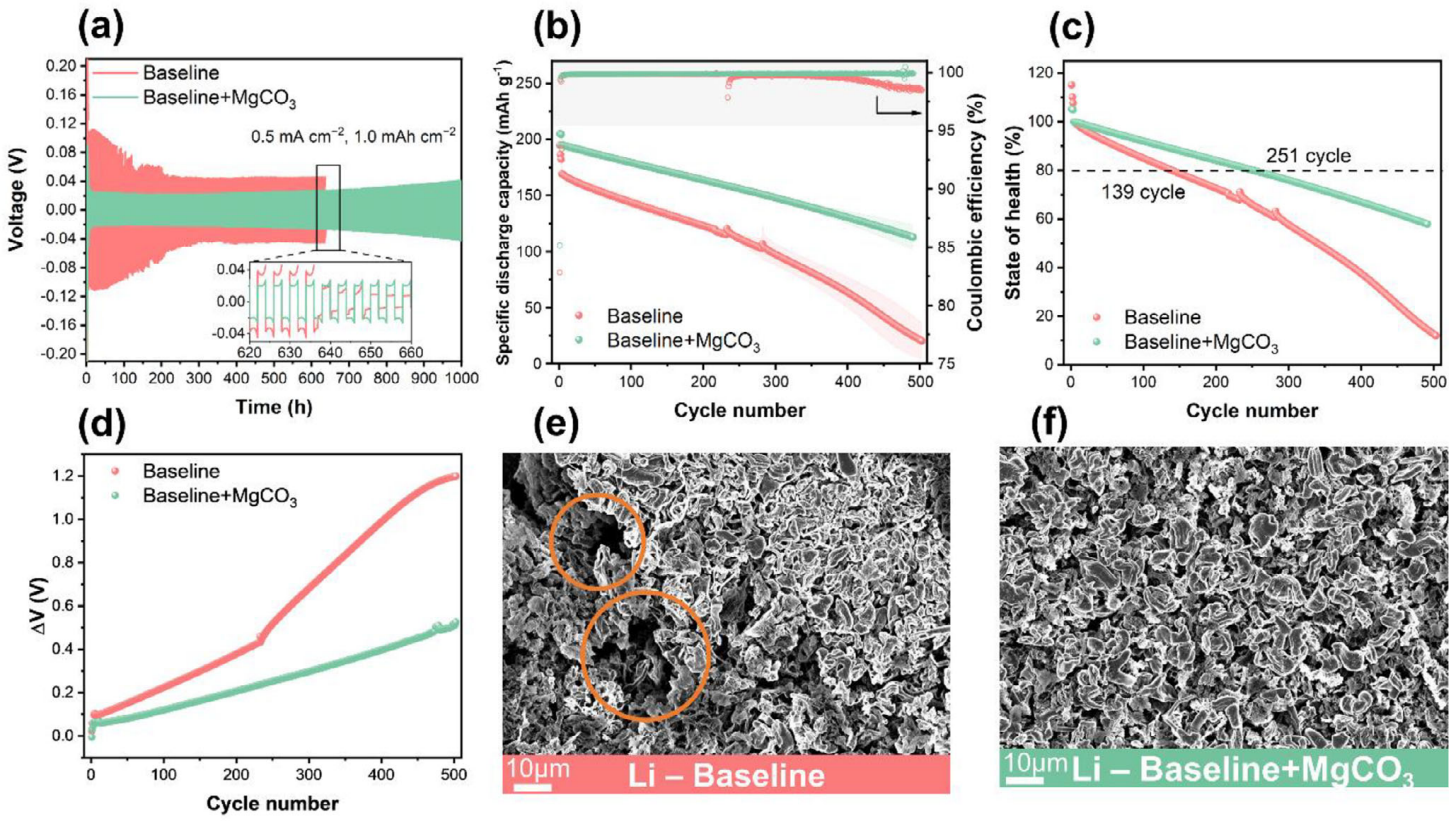
a) Voltage‐time profiles of Li||Li cells containing considered electrolytes. b) Specific discharge capacity versus cycle number curve of NMC811||Li cells using considered electrolytes, along with c) Capacity retention and d) Overvoltage versus cycle number plot. SEM images of the Li electrode collected after the 10^th^ cycle from NMC811||Li cells using e) Baseline electrolyte and f) Baseline+MgCO_3_ electrolyte.

The effectiveness of MgCO_3_ was further demonstrated by the galvanostatic cycling stability of Li||Li symmetric cells (**Figure** **2**a). With the baseline electrolyte, obvious voltage fluctuations were observed, which were accompanied by a drastic voltage drop after 640 h. This is attributed to a short circuit caused by Li dendrite penetration during the Li deposition and dissolution process. In contrast, Li||Li cells containing baseline+MgCO_3_ demonstrated considerably reduced overvoltage and voltage fluctuations. This extended the galvanostatic cycling stability and can be attributed to the formation of an effective SEI.

The electrochemical performance was further evaluated in a full cell setup by pairing a Li metal anode with an NMC811 cathode. The cells were galvanostatically cycled at a current rate of 1C with an upper cut‐off voltage of 4.3 V (Figure 2b). An initial specific discharge capacity of ≈195 mAh g^−1^ was observed for cells with baseline electrolyte at a current rate of 0.1 C, whereas a substantially higher specific discharge capacity of ≈205 mAh g^−1^ was observed for cells using MgCO_3_‐containing electrolyte, resulting in a specific discharge capacity difference (ΔQ) of ≈10 mAh g^−1^. ΔQ increased further to ≈25 mAh g^−1^ (≈169 mAh g^−1^ for baseline and ≈194 mAh g^−1^ for baseline+MgCO_3_) after formation cycles at a current rate of 1C. This discrepancy could be attributed to reduced resistance during Li deposition and dissolution in cells containing MgCO_3_, as evidenced by lower overvoltage (ΔV) observed in cells with baseline+MgCO_3_ compared to baseline counterparts (Figure 2d). In addition, cells containing MgCO_3_ electrolyte showed a higher initial CE (≈83.2% for baseline and ≈85.4% for baseline+MgCO_3_) and maintained superior overall CE (averaged from 5^th^ to 503^th^ cycle; ≈99.6% for baseline and ≈99.9% for baseline+MgCO_3_), indicating reduced parasitic reactions and effective SEI formation enabled by MgCO_3_. The beneficial effect of MgCO_3_ is further supported by improved galvanostatic cycling performance, which reached ≈251 cycles at 80% of the state‐of‐health (SOH_80%_) compared to cells with baseline electrolyte, which achieved only ≈139 cycles at SOH_80%_ (Figure 2c). The observed improvement can be attributed to the enhanced reversibility of Li metal deposition and dissolution during charge and discharge, which can be confirmed by the surface morphology of harvested Li metal electrodes from NMC811||Li cells. Galvanostatically cycled cells with the baseline+MgCO_3_ electrolyte show more homogeneous Li deposits, whereas cells with baseline electrolyte exhibit porous Li deposits on the Li metal surface (Figure 2e,f).

### 2.2 Functionality of MgCO_3_ Additive

**Figure 3:**
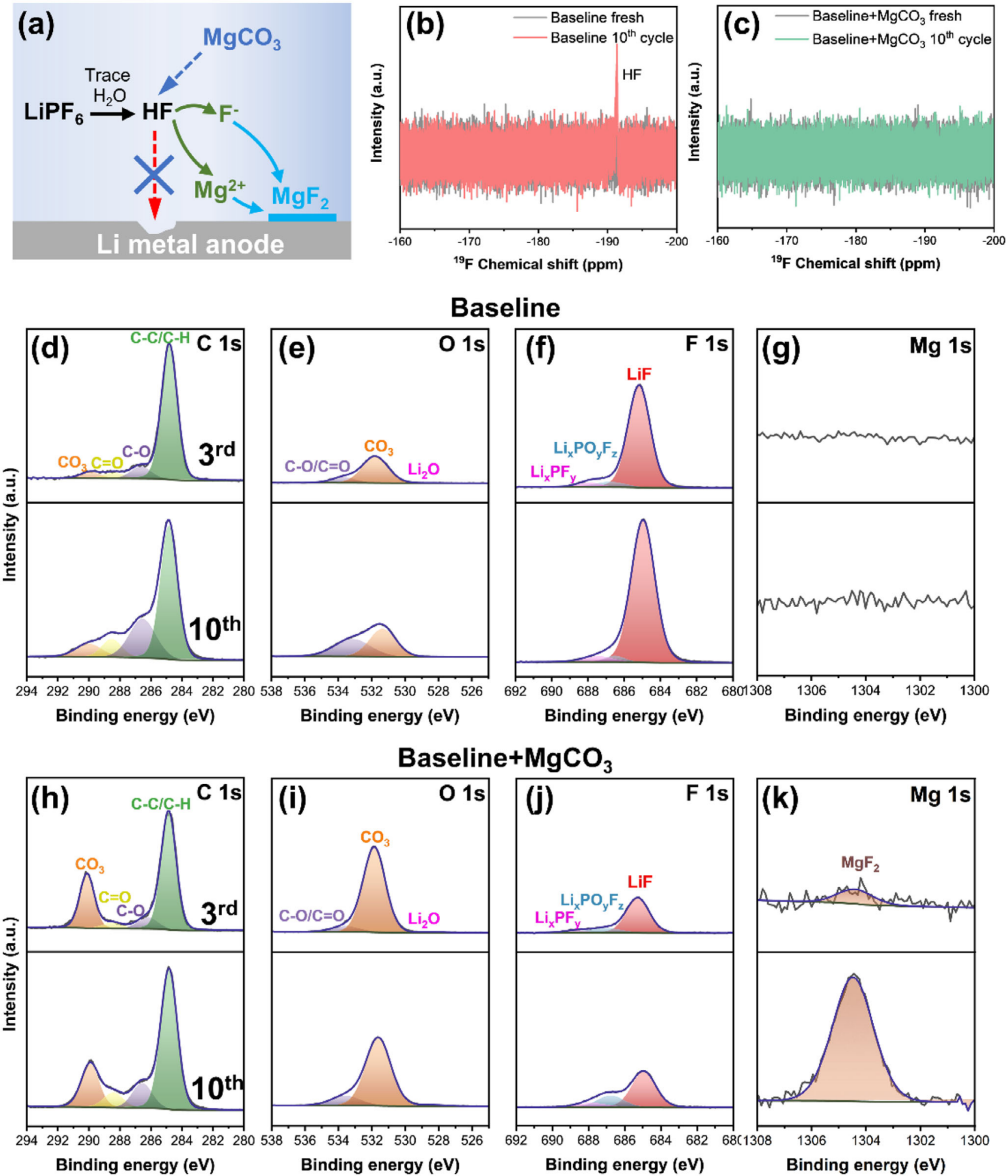
a) Illustration of the dissolution pathway of MgCO_3_. ^19^F NMR analysis results of electrolytes harvested from two NMC||Li cells after 10 cycles with b) Baseline and c) Baseline+MgCO_3_ electrolytes. Selected XPS spectra of d) C 1s, e) O 1s, f) F 1s, g) Mg 1s on Li electrodes harvested from NMC811||Li cells containing baseline electrolyte after 3 cycles (upper row) and 10 cycles (lower row). Selected XPS spectra of h) C 1s, i) O 1s, j) F 1s, k) Mg 1s on Li electrodes harvested from galvanostatically cycled NMC811||Li cells containing baseline+MgCO_3_ electrolyte after 3 cycles (upper row) and 10 cycles (lower row).

MgCO_3_ is hardly soluble in organic carbonate solvents, forming microparticles that remain suspended in the solution as a stable suspension (Figure S1, Supporting Information). However, PF_6_^−^ is susceptible to hydrolysis and continuously produces HF.^[^ ^23^ ^]^ HF serves as a proton donor and reacts with MgCO_3_ microparticles. This system allows MgCO_3_ to act as an HF scavenger while HF provides the necessary protons to dissolve the MgCO_3_ microparticles and release Mg^2+^ cations. The Mg^2+^ cations then move to the Li metal surface and form MgF_2_ with F^−^ after supersaturation, as illustrated in **Figure** **3**a. The ^19^F nuclear magnetic resonance (NMR) spectra of the harvested electrolytes demonstrated the effectiveness of MgCO_3_ as an HF scavenger (Figure 3b,c). The characteristic peak at ‐191.4 ppm was assigned to HF,^[^ ^24^, ^25^ ^]^ which only appeared in the baseline electrolyte spectrum after the 10^th^ cycle. Eliminating HF can effectively suppress the corrosion of Li metal and electrolyte decomposition.^[^ ^19^ ^]^ However, its impact on the reversibility of Li deposition and dissolution is less pronounced, as this process is primarily influenced by the effectiveness of the formed SEI.^[^ ^26^ ^]^

X‐ray photoelectron spectroscopy (XPS) was conducted to investigate the SEI composition on Li metal anode surface. Due to the high reactivity of Li metal, it is inevitable to form a native passivation layer (NPL) on the Li surface with trace amounts of oxygen, moisture, and lubricants during production and storage.^[^ ^27^, ^28^, ^29^, ^30^ ^]^ Therefore, pristine Li was included as a reference for comparison. As shown in Figure S2 (Supporting Information), the NPL on the pristine Li surface is characterized by peaks at 289.9 eV in the C 1s spectra and 531.6 eV in the O 1s spectra, which are attributed to lithium carbonate (Li_2_CO_3_), a common species in the NPL. In addition, organic components are also observed, as confirmed by the C═O (288.5 eV), C─O (286.7 eV), and C─C/C─H (284.8 eV) peaks in the C 1*s* spectra.

Distinct peaks are observed on the Li metal surface collected from cycled cells containing different electrolytes, highlighting variations in the SEI composition depending on the electrolyte formulation. For Li metal harvested from cells with a baseline electrolyte, a pronounced peak corresponding to C─C/C─H is observed in the C 1s spectra after the 3^rd^ cycle, along with organic compounds of C═O and C─O (Figure 3d). However, the CO_3_ peak, which is the main species for NPL, exhibited minor intensity, as confirmed in the O 1s spectra (Figure 3e). Higher amounts of organic components together with lower amounts of Li_2_CO_3_ suggest modification of the NPL and formation of an organic‐rich SEI. In addition, an intensive peak of LiF (684.8 eV) is observed in the F 1s spectra (Figure 3f). LiF is generally considered a crucial component of an effective SEI on Li metal.^[^ ^19^ ^]^ However, the source and morphology of LiF within the SEI considerably impact its effectiveness.^[^ ^31^ ^]^ For LiPF_6_‐based electrolytes, the formation of LiF is controlled by the transformation of HF and the reduction of PF_6_^−^ anions.^[^ ^32^, ^33^, ^34^ ^]^ The transformation of HF, i.e., HF reacting with Li metal, is an uncontrolled process that can lead to the formation of a thick and non‐uniform SEI, which may not effectively facilitate the Li^+^ ion transport and could lead to the formation of dendritic Li.^[^ ^35^, ^36^, ^37^ ^]^ Consequently, this contributes to increased interfacial resistance and reduced efficiency during charge and discharge.^[^ ^31^, ^36^, ^37^, ^38^, ^39^ ^]^ This phenomenon is corroborated by the observation of HF formation in NMR spectra and the decline in galvanostatic cycling performance in cells with baseline electrolyte. Extending to 10 cycles reveals a continued increase in the intensity of organic compounds and LiF in the XPS spectra, indicating further degradation of the electrolyte and Li metal (Figure 3d–f).

In contrast, the C 1s spectra of Li metal harvested from cells with a baseline+MgCO_3_ electrolyte show an increase in CO_3_ peak and a decrease in the C─C/C─H peak compared to the baseline electrolyte counterpart, suggesting a higher amount of Li_2_CO_3_ on the surface (Figure 3h) which could originate from the NPL. This is further supported by the similar O 1s spectra of Li metal harvested from cells containing baseline+MgCO_3_ electrolyte compared to pristine Li metal (Figure 3i). These observations suggest the formation of a thin SEI on the NPL or minor modification of the NPL by the electrolyte components. In addition, a noticeably weaker signal of LiF is observed in the F 1s spectra for Li metal with baseline+MgCO_3_ electrolyte (Figure 3j). The formation of LiF in this case can be considered to the reduction of PF_6_^−^ anions, as HF is effectively scavenged by MgCO_3_. After 10 cycles, the organic compounds on SEI remain largely unchanged, while an increase is observed for species stemming from PF_6_^−^ anions (LiF, Li_x_PF_y_, Li_x_PO_y_F_z_), as shown in Figure 3h–j and Figure S3 (Supporting Information). These observations indicate the formation of an effective SEI primarily composed of decomposition products resulting from the reduction of PF_6_^−^ anions, facilitated by the presence of MgCO_3_ as an additive. Although earlier research has centered on CEI stabilization in NMC111, both studies have noted that the consumption of PF_6_^−^ anions is increased by the use of Mg salt additives.^[^ ^40^, ^41^ ^]^ The preferential decomposition of lithium salt forms anion‐derived SEI with inorganic‐rich composites, which considerably enhances the galvanostatic cycling reversibility of Li metal electrodes.^[^ ^9^ ^]^ Furthermore, a MgF_2_ peak is detected in the Mg 1s spectra, with a notable increase in intensity during the charge and discharge cycles, indicating the gradual dissolution of MgCO_3_ microparticles (Figure 3k) and the potential formation of lithiophilic Li–Mg alloy through the further reaction of MgF_2_ with Li metal.^[^ ^42^, ^43^, ^44^ ^]^

In order to assess the individual contributions of Mg^2+^ and CO_3_^2−^ ions on electrochemical performance, equivalent molar amounts of Li_2_CO_3_, and magnesium hexafluorophosphate (Mg(PF_6_)_2_) were introduced into the baseline electrolyte. For comparison, the commonly used additives lithium bis(trifluoromethanesulfonyl)imide (LiTFSI) and magnesium bis(trifluoro‐methanesulfonyl)imide (Mg(TFSI)_2_) were also considered to evaluate the effectiveness of MgCO_3_. The galvanostatic cycling performance of NMC811||Li cells with these additives was systematically investigated. As shown in Figure S4a (Supporting Information), cells containing MgCO_3_, Li_2_CO_3,_ and Mg(PF_6_)_2_ exhibited higher initial discharge capacity compared to the cells with baseline electrolyte. However, cells containing Li_2_CO_3_ and Mg(PF_6_)_2_ exhibited faster capacity decay compared to cells containing MgCO_3_ (Figure S4b, Supporting Information). In contrast, cells containing Mg(TFSI)_2_ and LiTFSI showed lower initial specific discharge capacity, accompanied by a more pronounced capacity decay in cells containing Mg(TFSI)_2_ (Figure S4d, Supporting Information). This considerable drop is concomitant with elevated overvoltage in the first cycle, suggesting a discrepancy in the initial Li nucleation (Figure S4f, Supporting Information). Notably, either Li_2_CO_3_, Mg(PF_6_)_2_, LiTFSI, and Mg(TFSI)_2_ enabled improved capacity retention after 100 cycles in comparison to the baseline electrolyte counterpart. However, their efficacy was inferior to that of the electrolyte containing MgCO_3_ (Figure S4b, Supporting Information). Interestingly, while a similar trend in capacity drop was observed for cells with these additives, the overvoltage behavior is substantially different (Figure S4c,f, Supporting Information). A considerable increase in overvoltage was observed for cells with LiTFSI and Li_2_CO_3_ during continued cycles, whereas a slow increase was observed for cells with Mg(TFSI)_2_ and Mg(PF_6_)_2_. Notably, the cells with MgCO_3_ showed the lowest overall overvoltage over the cycles, indicating that both Mg^2+^ and CO_3_^2−^ ions are indispensable for the considered cell chemistry. In summary, the addition of MgCO_3_ serves two distinct functions: on one side, the CO_3_^2−^ anion acts as an HF scavenger. On the other side, the presence of Mg^2+^ cations accelerates the decomposition of PF_6_^−^ anions, leading to the formation of an anion‐derived SEI at the Li metal|electrode interface.

### 2.3 Electric Double Layer Behavior

In order to achieve a more profound comprehension of the underlying mechanism behind the accelerated decomposition of PF_6_^−^ in the presence of Mg^2+^ cations, it is important to analyze the behavior of the EDL. Considering that Li metal tends to lose electrons and release Li^+^ ions, the electrons remain near the metal surface, creating a net negative charge. This accumulation of negative charges on the Li metal electrode leads to an enrichment of cations and a deficiency of anions within the EDL region. Consequently, the components that directly coordinate with cations are predominantly present in the EDL and are reduced at the electrode|electrolyte interface to form the SEI.^[^ ^14^ ^]^ This understanding suggests that an MVC with a stronger binding affinity for anions can potentially attract more anions into the EDL compared to monovalent Li^+^ ions. It has been theoretically and experimentally demonstrated that Cu^2+^ cations have a stronger binding with NO_3_^−^ anions than Li^+^ cations, which brought the Cu^2+^‐NO_3_^−^ complex to the electrode surface and formed an effective SEI.^[^ ^13^, ^14^ ^]^ Furthermore, finite element simulation from a previous study indicated that divalent cations, due to their higher charge state, are more likely to accumulate in the EDL than their monovalent counterparts.^[^ ^14^ ^]^ Thus, it is reasonable to assume that Mg^2+^ cations may also play a similar role.

**Figure 4:**
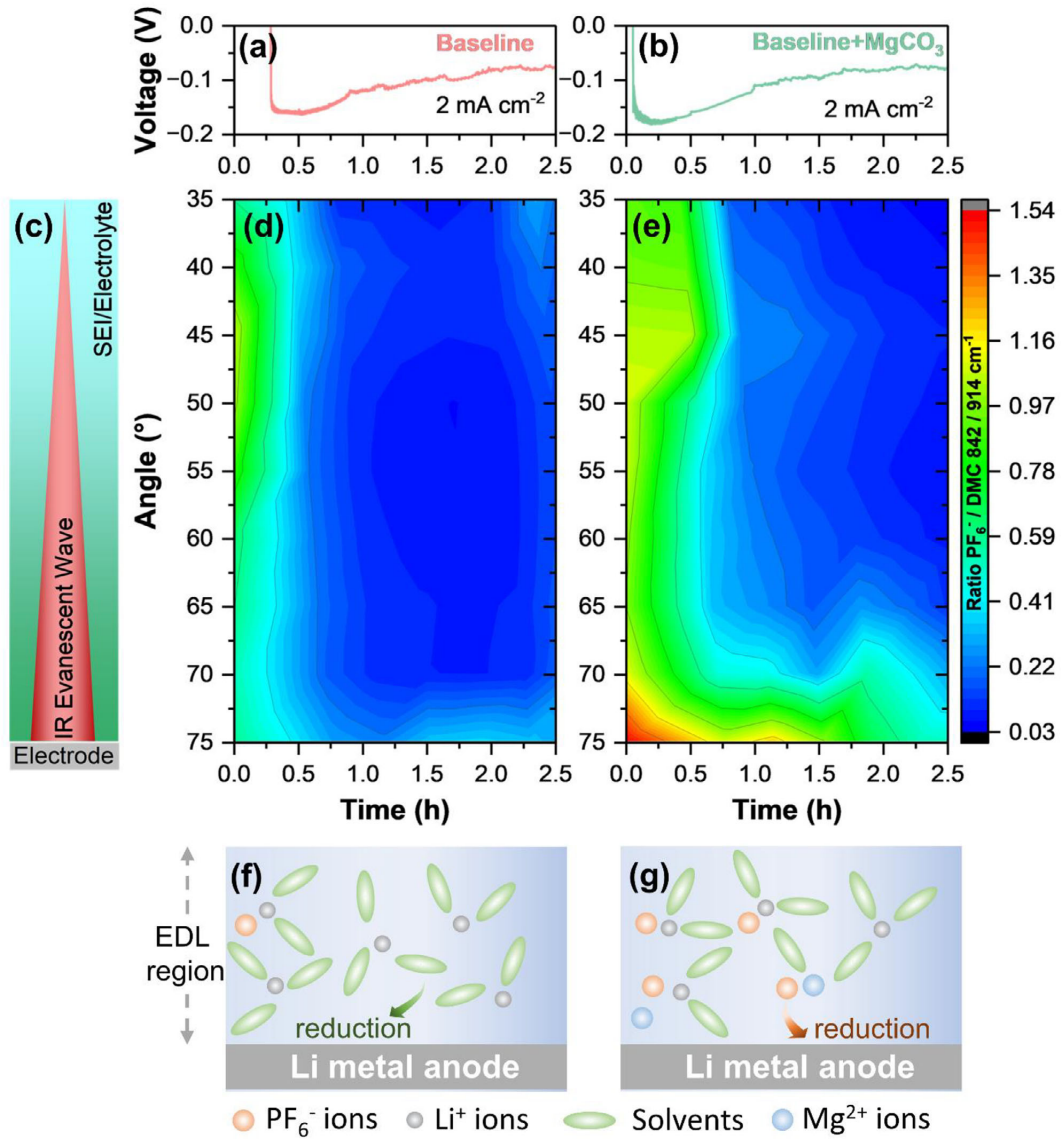
Voltage versus time profiles of the Li plating process evaluated in the Cu‐mesh@Si‐wafer||Li spectroelectrochemical cell with 1 M LiPF_6_ in DMC electrolyte a) without and b) with 0.1 m MgCO_3_ additive at a current density of 2 mA cm^−2^ with respect to the Li metal counter electrode. c) The angle represents the distance between the focus of the IR beam on the electrode surface and the bulk electrolyte. *Operando* ATR‐FTIR spectroscopy heatmap of the ratio of the ν(P‐F) band at 846 cm^−1^ of PF_6_^−^ to the ν(O‐CH_3_) band at 914 cm^−1^ of DMC during the Li plating at different angles d) without and e) with MgCO_3_ as electrolyte additive. Illustration of the EDL behavior during Li plating of electrolyte components f) without and g) with MgCO_3_.

*Operando* attenuated total reflectance‐Fourier transform infrared (ATR‐FTIR) spectroscopy was performed to understand the EDL behavior on the Li metal surface in different electrolytes. This technique enables the monitoring of molecular vibrations of species at the electrode|electrolyte interface and the electrolyte, such as the ν(P‐F) vibration of PF_6_^−^ anions. As the evanescent wave penetrates through the electrode in the spectro‐electrochemical cell, this method is very sensitive to the evolving interphases.^[^ ^45^ ^]^ By adjusting the incident angle, one can control the penetration depth of the evanescent wave, as the incident angle is inversely proportional to the penetration depth. With a higher incident angle, the penetration depth of the evanescent wave is closer to the SEI, whereas lower angles include more bulk electrolyte, as illustrated in Figure 4c. In the *operando* mode, this enables real‐time tracking of interphase depth profiles under varying conditions, such as different cell voltages or current densities. Here, the accumulation of the PF_6_^−^ anion at the interface was specifically investigated by monitoring the ν(P‐F) vibration ≈846 cm^−1^ using *operando* ATR‐FTIR spectroscopy, shown in **Figure** **4**.

The voltage profiles in Figure 4a,b of Li plating on the copper mesh exhibit analogous overvoltages for Li nucleation of ≈180 mV with and ≈160 mV without MgCO_3_. As Li plating progresses, the overvoltages of plating with both electrolyte formulations decrease to below 90 mV. The ratios of the band at ≈846 cm^−1^ (ν(P‐F), PF_6_^−^) with respect to the band at ≈914 cm^−1^ (ν(O‐CH_3_), DMC)^[^ ^46^ ^]^ (P/D) are monitored as a function of the incident angle and plating time (Figure 4d,e). As demonstrated in the initial stages of Li metal plating in the absence of MgCO_3_, the ratio of PF_6_^−^ to DMC drops to below 0.5 at all incident angles. With persistent Li plating, the P/D ratio further reduced to below 0.3 after 0.25 h, suggesting a relative depletion of PF_6_^−^ anions in respect to DMC molecules. The observations indicate that only a limited amount of PF_6_^−^ anions are accumulated within the EDL region, and that the EDL region is mostly composed of DMC solvent, as illustrated in Figure 4f. This leads to the formation of solvent‐derived SEI with organic‐rich compounds, which has been shown to adversely impact the Li metal deposition and dissolution process.

In the presence of MgCO_3_, the initial P/D ratio is considerably higher than that without MgCO_3_, especially at incident angles >70° (Figure 4e). As the penetration depth of the spectra recorded at 75° incident angle is the lowest, this region corresponds to the area adjacent to the electrode surface. Thus, the evidence suggests an accumulation of PF_6_^−^ anions at the electrode|electrolyte interface, especially within the EDL on the Li surface. With persistent Li metal plating, the P/D ratio is slightly reduced but remains >0.7 during the Li plating process. This trend suggests that, although the amount of PF_6_^−^ anions at the Li metal surface is gradually reduced with ongoing Li plating, the continuous release of Mg^2+^ cations facilitates the ongoing accumulation of PF_6_^−^ anions into the EDL region from the bulk electrolyte (Figure 4g). The accumulation of PF_6_^−^ promotes its preferential reduction of PF_6_^−^, resulting in the formation of anion‐derived SEI, which can effectively protect the Li metal surface.

## 3 Conclusion

In summary, we successfully demonstrated an MVC approach for forming an anion‐derived SEI on Li for LMBs by strategically selecting MgCO_3_ for its dual functionality: i) CO_3_^2−^ scavenges HF, and ii) gradual dissolution releases Mg^2^⁺, which enriches anions in the EDL and drives their preferential reduction. *Operando* ATR‐FTIR spectroscopy analysis confirmed the accumulation of PF_6_^−^ anions within the EDL on the Li surface during the plating process. Such anion‐derived SEI displays superior interfacial charge transfer kinetics, reducing the overvoltage and promoting homogeneous and dense Li metal deposits. Moreover, a substantially enhanced galvanostatic cycling performance was observed in NMC811||Li cells using MgCO_3_‐containing electrolytes, achieving ≈251 cycles at SOH_80%_ compared to only ≈139 cycles for cells containing the baseline electrolyte. This MVC approach demonstrated compatibility with commercial organic carbonate‐based electrolytes and high‐Ni cathodes, relying on a low loading of the abundant and inexpensive salt MgCO_3_, thereby reducing reliance on costly specialty electrolyte additives. In summary, this methodology provides a pragmatic and scalable approach for engineering anion‐derived SEI using the MVC strategy while reducing both the production cost and the environmental impacts associated with LMBs.

## 4 Experimental Section

### Materials

Battery‐grade dimethyl carbonate (DMC), lithium hexafluorophosphate (LiPF_6_), and lithium bis(trifluoromethanesulfonyl)imide (LiTFSI) were purchased from E‐Lyte Innovations GmbH with a purity of >99%. Fluoroethylene carbonate (FEC), lithium carbonate (Li_2_CO_3_), and zinc chloride (ZnCl_2_) were purchased from Sigma–Aldrich, and magnesium carbonate (MgCO_3_) was purchased from Acros Organics, all with a purity of >99%. Magnesium bis(trifluoromethanesulfonyl)imide (Mg(TFSI)_2_) was purchased from Solvionic with a purity of 99.5%. Magnesium hexafluorophosphate (Mg(PF_6_)_2_) was purchased from Sagechem with a purity of 99%.

All chemicals were used as received. LiPF_6_ was dissolved in the FEC:DMC (1:4 by volume) solvent mixture to formulate 1M LiPF_6_‐based electrolyte (referred to as baseline). The amount corresponding to 0.05 M MgCO_3_ was added into the baseline electrolyte to form a suspension (baseline+MgCO_3_) (Figure S1, Supporting Information).

### Electrodes and Cell Assembly

Li metal (150 µm thickness) foil and Cu foil were purchased from Albemarle and Fisher Scientific GmbH, respectively. Calendered NMC811 single‐sided coated electrode sheets with an active mass loading of 5.4 mg cm^−2^ were provided by Umicore. For Li||Li symmetric cells and Li||Cu cells, the Li metal and Cu foils were punched into ø12 mm and ø15 mm disks, respectively. For NMC||Li cells, the NMC811 electrodes were punched into ø14 mm disks and subsequently dried at 120 °C under vacuum (<10^−2^ mbar) for 12 h, whereas the Li metal was punched into ø15 mm disks. Celgard 2500 membrane was used as a separator for all measurements and punched into ø16 mm disks. All materials were stored in an argon‐filled glovebox before cell assembly.

CR2032 coin cells were assembled in an argon‐filled glovebox using a stack of a negative electrode, a layer of separator, and a positive electrode, where the stainless steel (SUS) spacers of 0.5 mm and 1 mm thickness were combined with a metal spring of 1.5 mm height to ensure enough pressure and contact. The cells were pressed with an automatic coin cell crimper (Hohsen), applying a pressing force of 7 kN for 5 s. The volume of electrolyte was set to 30 µL.^[^ ^47^ ^]^

### Electrochemical Performance Evaluation

For Li||Cu and Li||Li cells, the Li deposition and dissolution cycling stability was evaluated at 0.5 mA cm^−2^ and 1.0 mAh cm^−2^ Li deposition. For NMC||Li cells, a constant current‐constant voltage (CC‐CV) charge and constant current discharge protocol was applied in a voltage range from 3.0 to 4.3 V. The cell formation conditions comprised 3 cycles at 0.1C. The cells were then galvanostatically cycled for 500 cycles at 1C to evaluate the galvanostatic cycling performance. All cells were evaluated on a battery tester (MACCOR series 4000) at 20 °C.

### Scanning Electron Microscopy

Li and Cu metal surfaces were investigated via scanning electron microscopy (SEM) after Li plating. The cells under consideration were opened in an argon‐filled glovebox, and the samples were rinsed with DMC (1 mL) to remove the residual electrolyte. Samples were transferred to the SEM chamber using a vacuum‐sealed sample holder and analyzed using an Auriga CrossBeam workstation (Zeiss) at an acceleration voltage of 3 kV and a working distance of 5 mm using the InLens detector.

### Nuclear Magnetic Resonance Spectroscopy

Nuclear magnetic resonance spectroscopy (NMR) measurements were used to detect the formation of HF in electrochemically evaluated cells. To get enough electrochemically treated electrolyte, 3 cells were disassembled after considering cycles and immersed in 600 µL C_6_D_6_ solvent to extract the electrolyte. The solutions were filtered with a 0.22 µm syringe filter and then transferred into an NMR‐tube. The fresh electrolyte was also injected into a C_6_D_6_ containing NMR‐tube and used as a reference. The NMR measurements were carried out with a 400 MHz AVANCE III HD spectrometer (Bruker).

### Operando Attenuated Total Reflection Fourier‐Transform Infrared (ATR‐FTIR) Spectroscopy

*Operando* ATR‐FTIR measurements were carried out on an Invenio‐R (Bruker, US) with a mercury‐cadmium‐telluride (MCT) detector on a ZnSe ATR crystal. A VeeMAX III specular reflection accessory (Pike Technologies, US) was used for automatic angle variation in combination with an in‐house‐built spectro‐electrochemical cell. Details about the spectro‐electrochemical cell setup and assembly were reported previously.^[^ ^48^ ^]^ The spectra were obtained continuously by accumulating 32 sample and 32 background scans with a spectral resolution of 4 cm^−1^ at incident angles in 5° steps from 35° to 75°. For background scans, a second cell without electrolyte was inserted into the spectrometer. All spectra were processed with H_2_O correction. Additionally, concave rubber band background correction was carried out with 15 iterations to increase comparability at different incident angles. The absorbance intensities at 846 and 914 cm^−1^ were used for the ratio calculation (ν(P‐F) from PF_6_^−^ and ν(O─CH_3_) from DMC).

The spectroelectrochemical cell was assembled according to a previous study.^[^ ^48^ ^]^ A prime CZ‐Si (100) double‐side polished p‐type (Boron‐doped) wafer with 1–10 Ω∙cm resistance and total thickness variation <10 µm (MicroChemicals, Germany) was pressed against the reflective plane of a ZnSe semisphere ATR crystal (25 mm diameter, Bruker, US) with an O‐ring and fixed by clamping the cell housing with six screws. Before clamping, Nujol oil (VWR International, US) was added between the Si wafer and ZnSe surface. A round copper mesh (30 mm diameter) with a 5 cm strip, to connect cables via crocodile clips, was used as a current collector. Afterward, the glass fiber separator (Whatman GF/D), 300 µL electrolyte, Li metal (13 mm diameter, 500 µm thick) as a counter electrode, and the current collector with spring and gaskets were sandwiched and clamped with an external additional screw. Electrochemical performance evaluation was performed with an Autolab PGSTAT204 (Metrohm, Germany) controlled by the NOVA 2.1 software. After a 1 h open circuit voltage (OCV) step, a current density of 2 mA cm^−1^ (with respect to the Li metal counter electrode) was applied, and voltage was monitored. The IR measurements were carried out during cell operation. The IR spectrometer was located in a box with a nitrogen atmosphere and was constantly flushed with nitrogen. As electrolytes 1 m LiPF_6_ in DMC with and without 0.1 m MgCO_3_ were used.

### X‐Ray Photoelectron Spectroscopy

To prevent exposure to oxygen and moisture, the X‐ray photoelectron spectroscopy (XPS) samples were sealed in vials after preparation. The sealed vials were opened in an argon‐filled glovebox connected to the instrument shortly before measurement. XPS measurements were performed on an Axis Ultra DLD spectrometer (Kratos Analytical, UK) equipped with a monochromatic Al K_α_ source (E_photon_ = 1486.6 eV) at a 0° angle of emission using a 10 mA emission current and an accelerating voltage of 12 kV for the X‐ray source. The hemispherical analyzer was set to a pass energy of 20 eV. The analyzed area was ≈700 µm x 300 µm (“hybrid” lens mode, “slot” aperture), and a charge neutralizer was utilized to compensate for sample charging. During measurements, the pressure within the analysis chamber was always lower than 1∙10^−7^ mbar. Sputter depth profiling of the negative electrode was conducted using monoatomic argon at an energy of 500 eV. The depth profiling procedure consisted of two sputter cycles of 360 s each. Two samples of each type (pristine samples, samples which had been in contact with electrolyte, formatted samples, and cycled samples) were measured to ensure reproducibility. For peak fitting, the CasaXPS software (Casa Software, UK) was used^[^ ^49^ ^]^ and the peaks were assigned based on known literature values.^[^ ^27^, ^28^, ^29^ ^]^ The energy scale in the spectra was adjusted using the C 1s peak at 284.8 eV (C‐C) as an internal reference.

### Statistical Analysis

To ensure reproducibility, at least three nominally identical electrochemical analyses were performed on different cells for Li||Cu and NMC811||Li cells. The data were presented as the mean ± standard deviation (SD) using OriginPro 2020.

## Conflict of Interest

The authors declare no conflict of interest.

## Supporting information



Supporting Information

## Data Availability

The data that support the findings of this study are available from the corresponding author upon reasonable request.
