# Supplementary material for: Multi‐Valent Cation Strategies for Controlling Interphase Chemistry at the Lithium Metal Anode
Source: Small Methods. 2025 Sep 18;9(11):e01449. doi: 10.1002/smtd.202501449 (PMC12641351; doi:10.1002/smtd.202501449)
Supplement: Supplementary file 1 — Supporting Information [file SMTD-9-e01449-s001.docx]

Supporting Information

**Multi-valent Cation Strategies for Controlling Interphase Chemistry at the Lithium Metal Anode**

*Peng Yan^**1^, Rui Xu^**1,3^, Matthias Weiling^1^, Bixian Ying^2^, Marian Cristian Stan^1^, Christian Wölke^1^, Masoud Baghernejad^1^, Jia-Qi Huang^3^, Martin Winter^1,2^, Peter Bieker^1*^, Isidora Cekic-Laskovic^1*^*

1: Helmholtz-Institute Münster (IMD-4), Forschungszentrum Jülich GmbH, Corrensstraße 48, 48149 Münster, Germany

2: MEET Battery Research Center, University of Münster, Corrensstraße 46, 48149, Münster, Germany

3: Advanced Research Institute of Multidisciplinary Science, Beijing Institute of Technology, Beijing, 100081, China

^*^Corresponding authors: i.cekic-laskovic@fz-juelich.de, p.bieker@fz-juelich.de

^**^These authors contributed equally


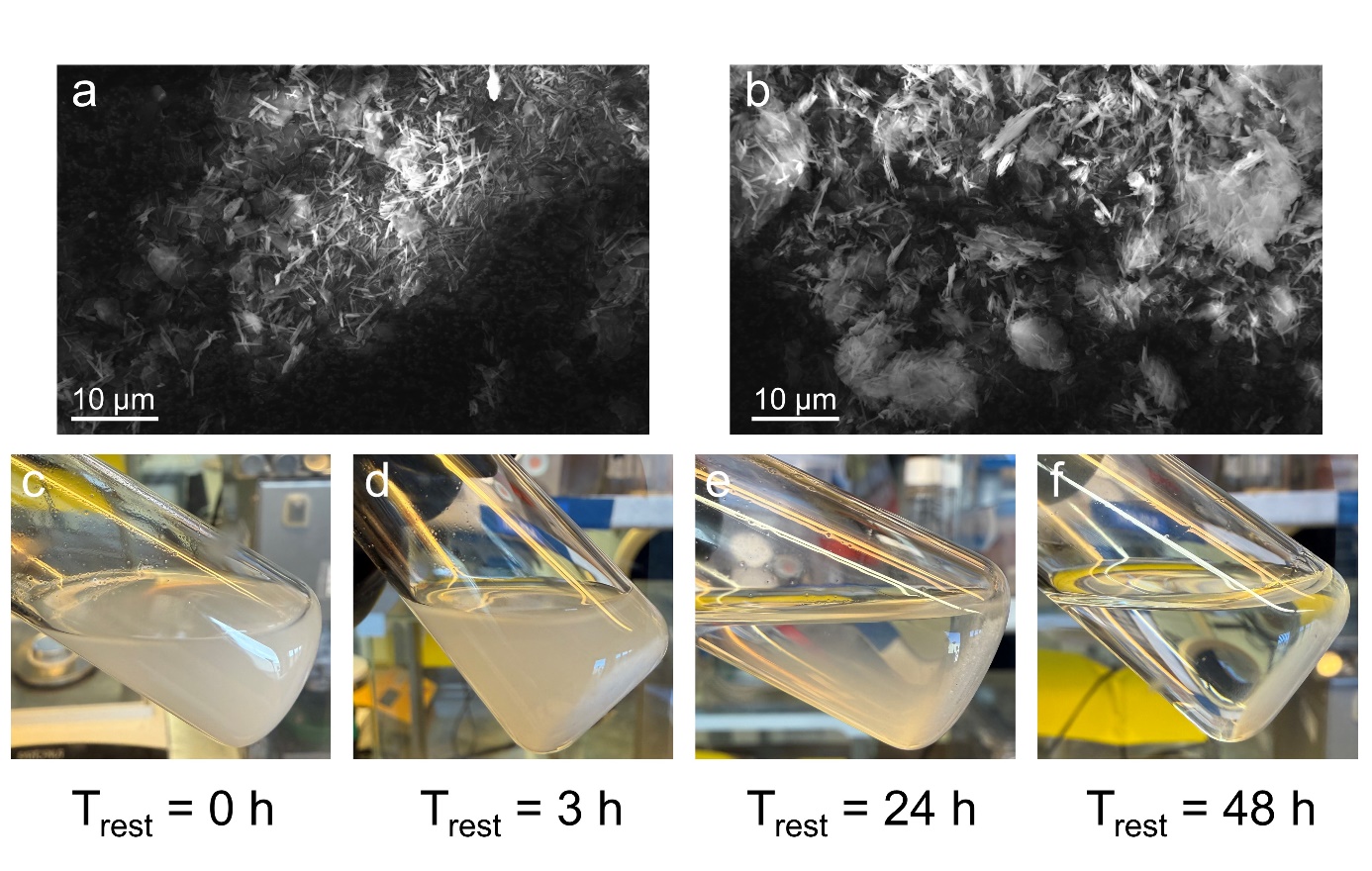


Figure S 1. (a)-(b) SEM images of MgCO_3_ powder. (c)-(f) Suspension state of the baseline electrolyte with 0.05M MgCO_3_ resting at different times.


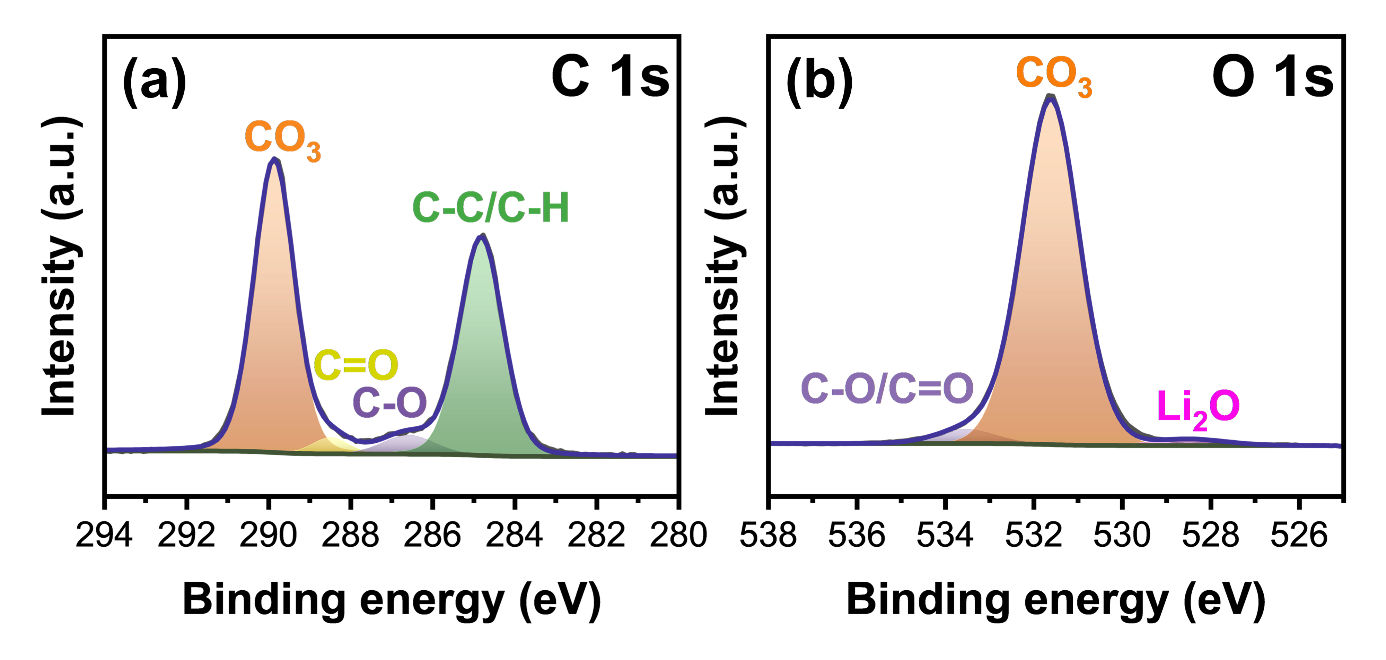


Figure S 2. Selected XPS core spectra of (a) C 1s and (b) O 1s on pristine Li electrode.


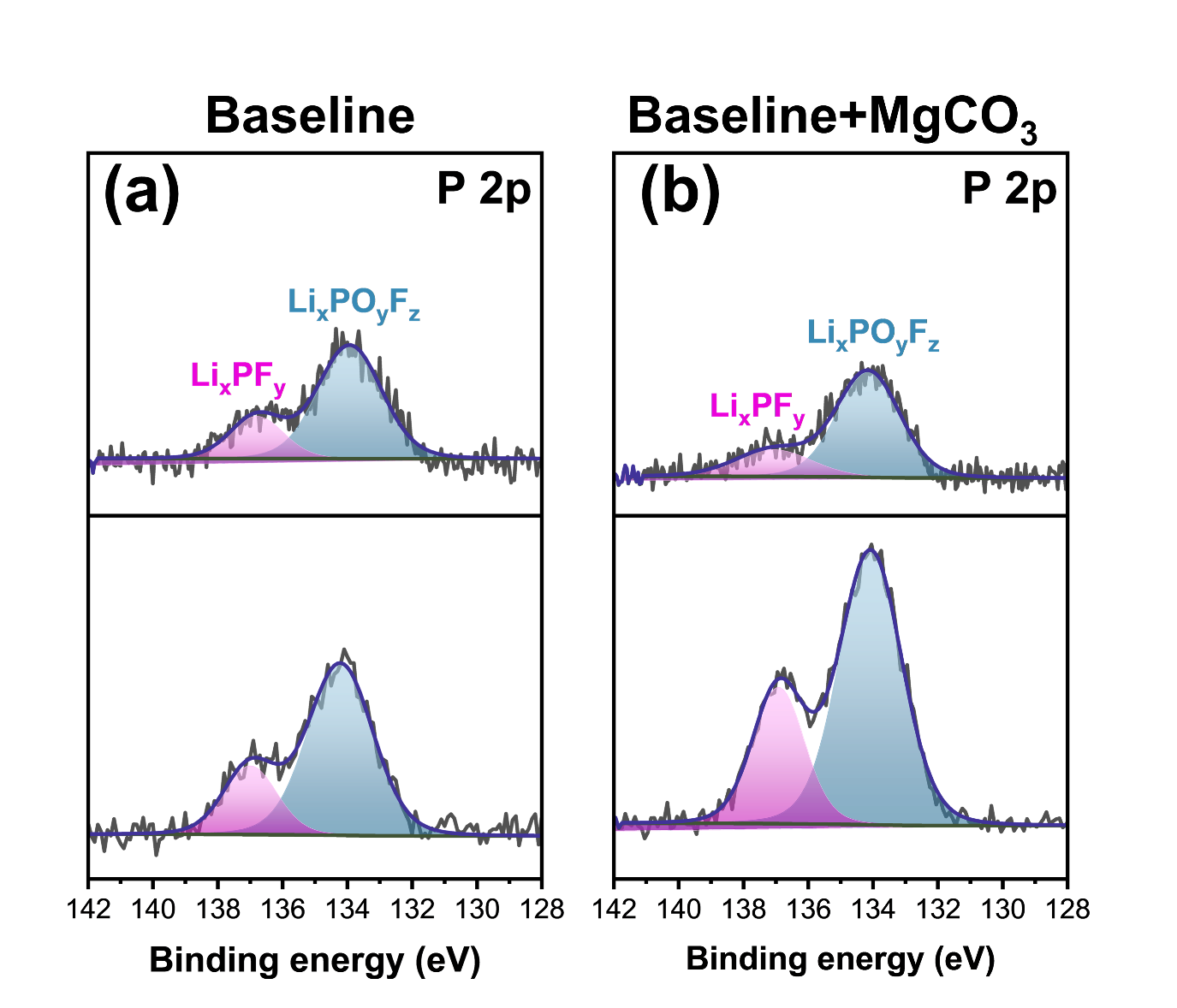


Figure S 3. Selected XPS core spectra of P 2p on Li electrodes harvested from NMC811||Li cells galvanostatically cycled using (a) baseline electrolyte and (b) baseline+MgCO_3_ electrolyte after 3 cycles (upper row) and 10 cycles (lower row).


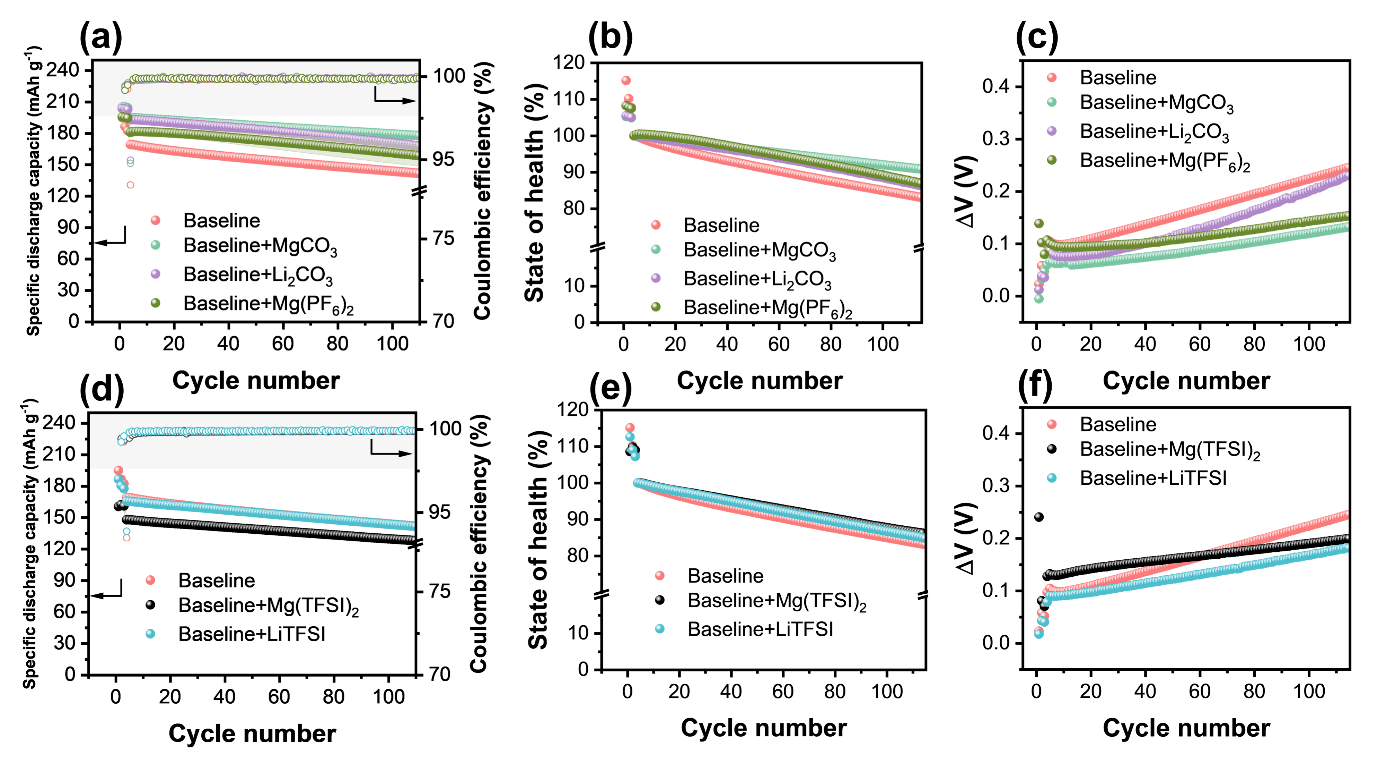


Figure S 4. (a) Galvanostatic cycling performance of NMC811||Li cells using MgCO_3_, Li_2_CO_3_ and Mg(PF6)_2_ containing electrolytes, along with (b) Capacity retention vs. cycle number curves and (c) Voltage vs. cycle number. (d) Specific discharge capacity vs. cycle number curves of NMC811||Li cells using Mg(TFSI)_2_ and LiTFSI containing electrolytes, along with (e) State of health and (f) Overvoltage vs. cycle number.


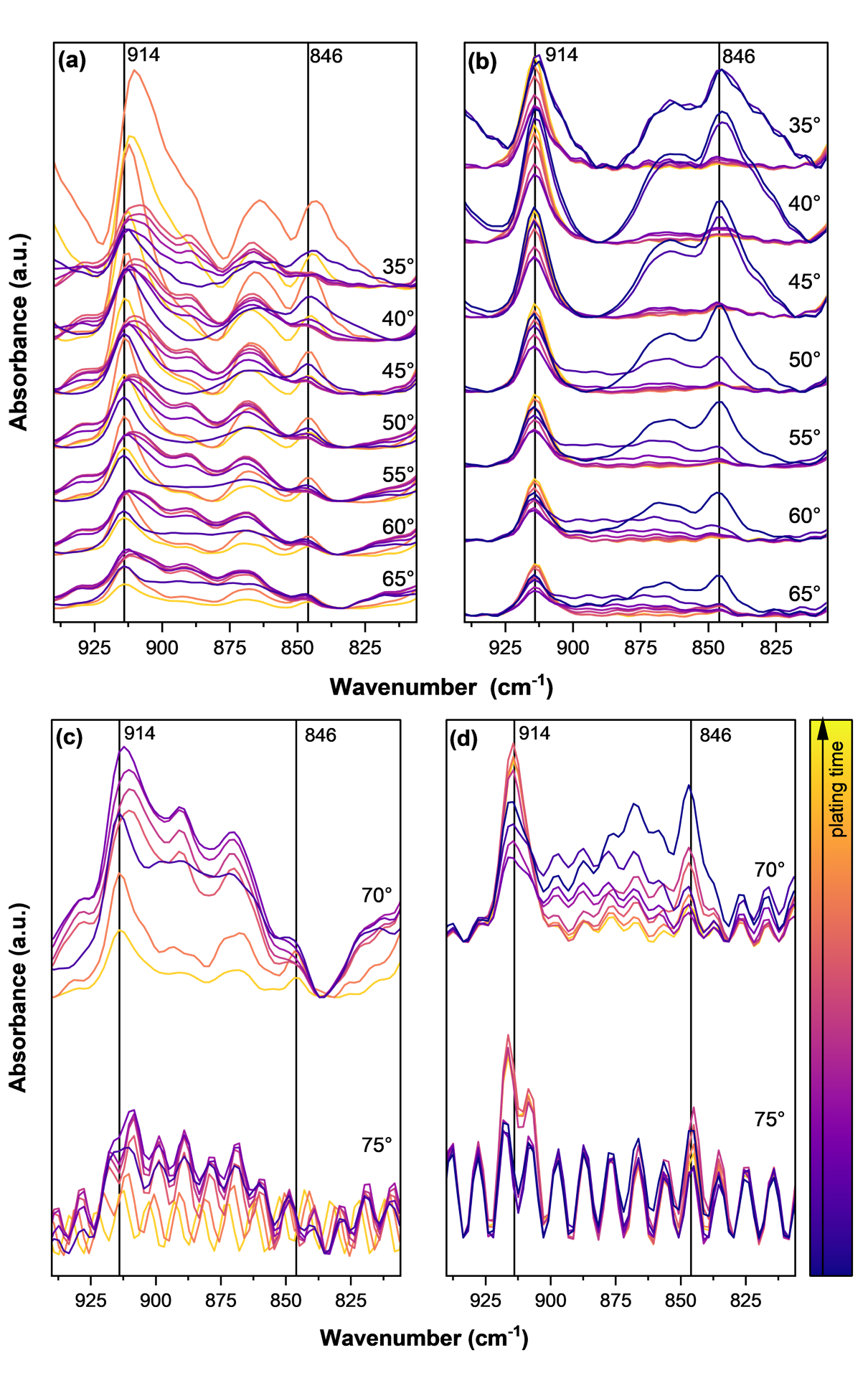


Figure S 5. Operando ATR-FTIR spectra recorded during the Li metal plating in the Cu-mesh@Si-wafer||Li spectro-electrochemical cell containing 1 M LiPF_6_ in DMC electrolyte a) and c) Without and b) and d) With 0.1 M MgCO_3_ additive at a current density of 2 mA cm^-2^ in respect to the Li metal counter electrode. The ν(P-F) band of PF_6_^-^ is positioned at ≈846 cm^-1^ and the ν(O-CH_3_) band of DMC at ≈914 cm^-1^

**Note 1: Potential Candidates for MVC strategy**

The Zn^2+^ and Mg^2+^ cations were selected as potential candidates due to their smaller ionic radius compared to Li^+^ (0.74 Å for Zn^2+^ and 0.72 Å for Mg^2+^). Both 0.05M ZnCl_2_ and 0.05M MgCO_3_ were evaluated as electrolyte additives in Li||Cu and NMC811||Li cell chemistries. Figure S 6a shows that Li||Cu cells with ZnCl_2_ additive present even higher Coulombic efficiency than those with MgCO_3_. However, the NMC811||Li cells with ZnCl_2_-containing electrolytes showed rapid capacity decay, dropping below 80% state of health after 25 cycles (Figure S 6b), and were therefore excluded for further investigation.


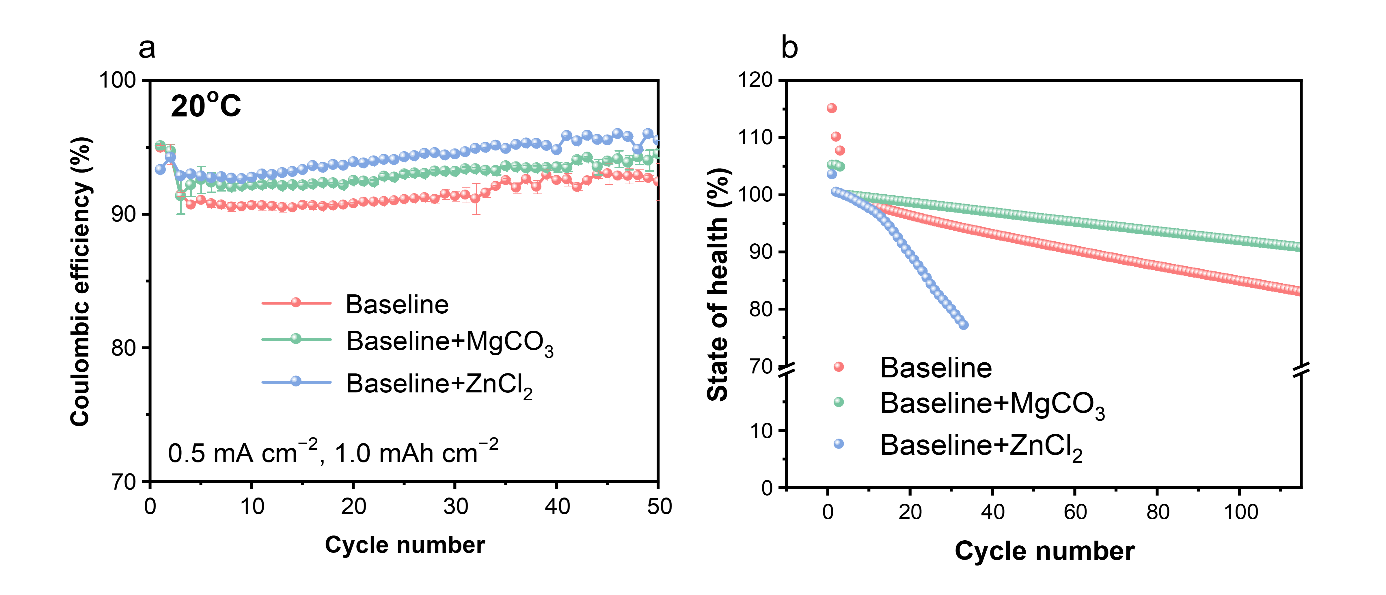


Figure S 6. Electrochemical performance of Li||Cu and NMC811||Li cells with considered electrolytes. (a) Coulombic efficiency of Li||Cu cells over the first 50 cycles. (b) Capacity retention vs. cycle number plot of NMC811||Li cells.
